# Zebrafish as a Model for Fetal Alcohol Spectrum Disorders

**DOI:** 10.3389/fphar.2021.721924

**Published:** 2021-12-15

**Authors:** Amena Alsakran, Tetsuhiro Kudoh

**Affiliations:** ^1^ Department of Biosciences, College of Life and Environmental Sciences, University of Exeter, Exeter, United Kingdom; ^2^ University of Exeter, Exeter, United Kingdom

**Keywords:** zebrafish, embryo, alcohol, ethanol, epiboly, fetal alcohol spectrum disorders

## Abstract

In this review, we will discuss zebrafish as a model for studying mechanisms of human fetal alcohol spectrum disorders (FASDs). We will overview the studies on FASDs so far and will discuss with specific focus on the mechanisms by which alcohol alters cell migration during the early embryogenesis including blastula, gastrula, and organogenesis stages which later cause morphological defects in the brain and other tissues. FASDs are caused by an elevated alcohol level in the pregnant mother’s body. The symptoms of FASDs include microcephaly, holoprosencephaly, craniofacial abnormalities, and cardiac defects with birth defect in severe cases, and in milder cases, the symptoms lead to developmental and learning disabilities. The transparent zebrafish embryo offers an ideal model system to investigate the genetic, cellular, and organismal responses to alcohol. In the zebrafish, the effects of alcohol were observed in many places during the embryo development from the stem cell gene expression at the blastula/gastrula stage, gastrulation cell movement, morphogenesis of the central nervous system, and neuronal development. The data revealed that ethanol suppresses convergence, extension, and epiboly cell movement at the gastrula stage and cause the failure of normal neural plate formation. Subsequently, other cell movements including neurulation, eye field morphogenesis, and neural crest migration are also suppressed, leading to the malformation of the brain and spinal cord, including microcephaly, cyclopia, spinal bifida, and craniofacial abnormalities. The testing cell migration in zebrafish would provide convenient biomarkers for the toxicity of alcohol and other related chemicals, and investigate the molecular link between the target signaling pathways, following brain development.

## Introduction

Ethyl alcohol (ethanol) is the most widely used and consumed drug in humans’ daily lives. Alcohol addiction became common in 1700s when the increased distillation of potent alcohol made vast quantities readily accessible to the masses (Lovely et al., 2016). During that time, some noted that the offspring of people who drank substantial quantities of distilled alcohol was sometimes small and weak, with a higher child mortality rate. Consequently, the research field and study of fetal alcohol spectrum disorder (FASD) syndrome were commenced. According to a survey, about 9.8% of pregnant women consume alcohol (ethanol) globally (Popova et al., 2019). The fetus of these women face an increased risk of lifelong fetal alcohol spectrum disorder (FASD) complications. FASD phenotypes are quite broad, and over 400 diseases can co-occur in FASD patients (Popova et al., 2016).

Alcohol poses a broad range of irreversible side effects on the human fetus. The maternal consumption of alcohol is considered to have a highly teratogenic effect on the fetus. No studies have reported a safe time period and quantity for alcohol intake during pregnancy. Its consumption has been widely reported to be teratogenic in all three trimesters of pregnancy. The higher consumption of alcohol during the first trimester is associated with brain and facial anomalies, whereas alcohol consumption during the second trimester can cause sponataneous abortions, and alcohol consumption during the third trimester is linked with reduced weight, brain volume, and height. Therefore, it could be concluded that the fetal exposure to alcohol at any point of pregnancy can cause irreversible damage and potentially lead to fetal alcohol spectrum disorders (FASD) and their related neurobehavioral deficiencies including the developmental and learning disorders (Easey et al., 2019; Popova et al., 2019; Reid et al., 2019). Women older than 30 years or with genetically slow alcohol metabolism are more likely to produce infants with FASD complications. The detrimental effects of alcohol on the CNS (central nervous system) are generally considered to be irreparable as alcohol not only decreases the volume of the brain but also damages the structures in the brain. Several researchers have reported smaller volumes of gray and white matter in the brains of individuals prenatally exposed to alcohol than controls (Bjorkquist et al., 2010; Nardelli et al., 2011; Roussotte et al., 2012). Prenatal exposure to alcohol affects the structure of the corpus callosum that eventually contains a smaller volume of white matter than normal (Lebel et al., 2011). Diffusion tensor imaging studies have also revealed disrupted white matter integrity and its relation to the behavior of individuals with prenatal exposure to alcohol (Riley et al., 2011). FASDs can cause craniofacial abnormalities and impairment of the central nervous system (CNS) production (Carvan et al., 2004; Kelly et al., 2009). The physical facial features that can indicate the FASD symptoms in an individual include a smooth philtrum, short palpebral fissures, and a thin vermillion border. Structural defects in the ocular, renal, cardiovascular, and auditory systems can also occur in children with prenatal exposure to alcohol. Microcephaly and prenatal or postnatal retarded growth is also quite common among children who were exposed to alcohol at birth. Consequently, the postnatal developmental and mental disorder are induced (Popova et al., 2019). Due to the apparent limitations of human trials, most research on FASD presently relies on translational animal models to discover the toxicity of ethanol in embryonic and fetal development, including rodent and zebrafish (Pinheiro-da-Silva and Luchiari, 2021). Rodent models, such as mice, have become an essential tool for investigating the effects of alcohol on all levels of development, especially as studies in humans and rodents show that blood alcohol content (BAC) has a similar influence on behavior across the species (Driscoll et al., 1990). As seen in humans, microcephaly and neuronal loss effect were demonstrated on the lab mice with some variation depending on strains (Bonthius et al., 2002; Ogawa et al., 2005; de Licona et al., 2009). Mice have been widely used as a model for FASD, however there are some disadvantages (Patten et al., 2014). Firstly, as mice embryos develop internally, it’s difficult to observe the early stages of development. This also makes live embryo analysis challenging. In addition, when using mammals, it is more difficult to determine the drug dosage and duration of exposure that will result in specific phenotypes. This is due to the mother’s metabolic processes needing to be considered and that the drug’s effects can only be detected after delivery. Besides, alcohol and drug testing are intrusive and may create stress to the mother, which might affect the findings (Pinheiro-da-Silva and Luchiari, 2021).

As an alternative model, zebrafish have also been used to study. Embryo development in normal and disease conditions due to their high fertility, size, and external development of the transparent embryo, which can be visualized easily. These features of zebrafish can be crucial for defining key times of ethanol exposure and allowing easy analyses of tissue morphogenesis, cell movement, and gene expression (Bilotta et al., 2002). Recent work from zebrafish revealed that ethanol can affect the early development of embryos in cell fate specification and cell migration at the gastrula stage, far before the stage of brain and craniofacial development and consequently cause the severe symptom of FASD at later stages of embryos (Blader and Strähle, 1998; Shan et al., 2015; Yelin et al., 2005; Zhang et al., 2010).

## Zebrafish as a Model for Studying Embryonic Brain Development

**FIGURE 1:**
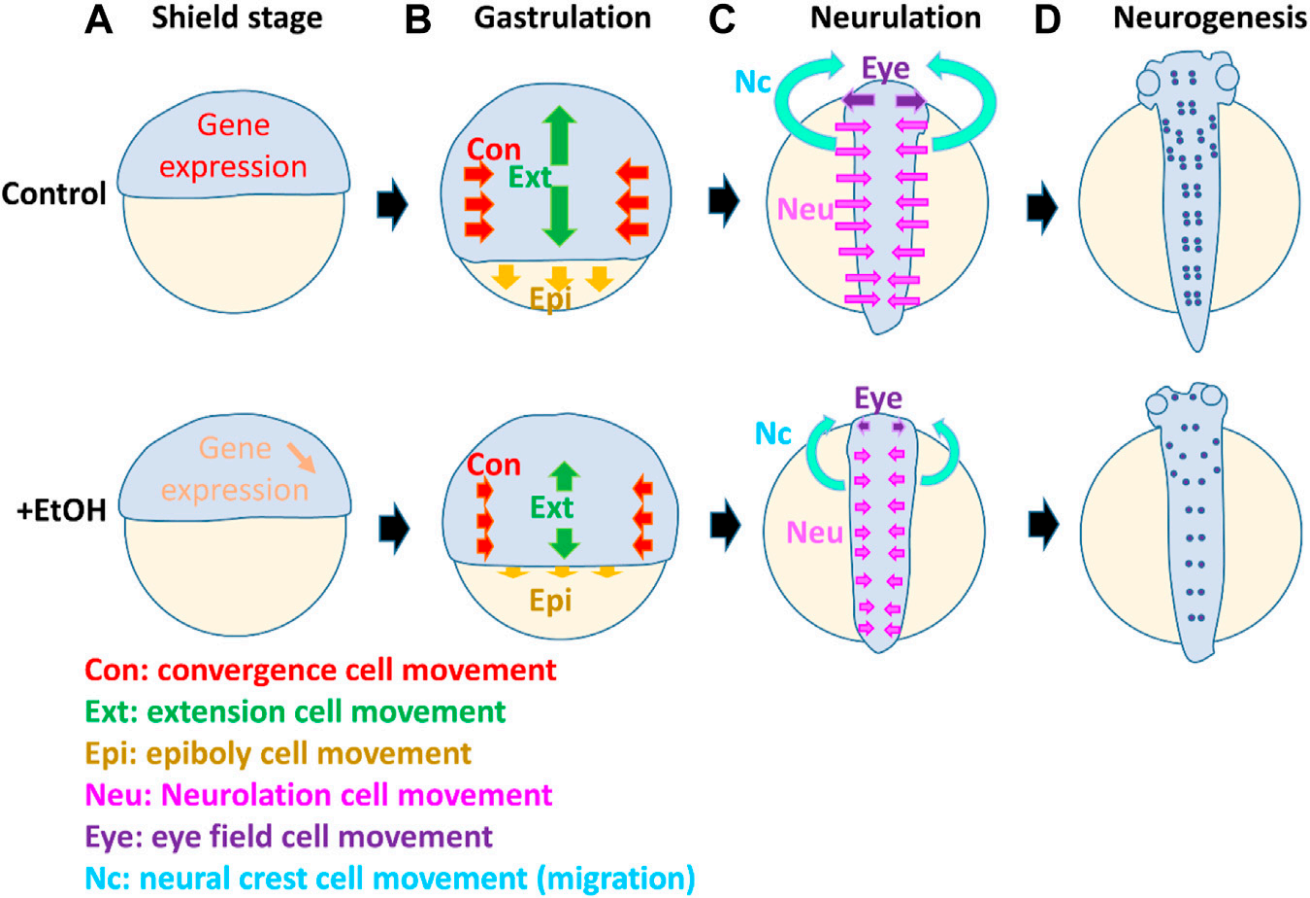
Model suggesting multiple targets of ethanol which affect cell differentiation, cell movement, and morphogenesis during different stages in the early embryo development. The diagram shows zebrafish embryos in normal development and compromised development in the presence of ethanol. At the onset of the gastrula stage [shield stage, **(A)**], the gene expressions (e.g., sox2 crucial for stem cells and CNS development) are suppressed by ethanol (downward arrow). During gastrulation **(B)**, ethanol affects the cell movement of convergence (Con, red arrows), extension (Ext, green arrows), and epiboly (Epi, yellow arrows). Smaller arrows in the ethanol-exposed embryo indicate the reduced distance of these cell movements. Subsequently, during neurulation **(C)**, ethanol affects the neurulation cell movement (Neu, pink arrows), by which the flat neural plate cells move to the midline and form a neutral tube. Reduced distance of the neurulation cell movement (pink arrows) causes incomplete closure of the brain and spinal cord. At the neurulation stages, cell movement also occurs in the eye field and neural crest. The single eye field (Eye) moves to the left and right, separating and forming two eyes. Neural crest cells which are derived from the side of the neural plate migrate anteriorly and form craniofacial structures. These eye field and neural crest cell movement (purple and light blue arrows, respectively) are also suppressed by alcohol, causing reduction of the distance of two eyes and craniofacial malformation. At the neurogenesis stage **(D)**, the number of neurons (purple dots) is reduced by ethanol, and axon growth is perturbed.

The zebrafish brain development initiates from the process of neural induction during the gastrula stage of the embryo. At this stage, two neural inducers represented by chordin and fibroblast growth factor (FGF) emanate from the dorsal organizer and marginal mesoderm, inducing the fate of the central nervous system (CNS) in the dorsal- and ventral-vegetal ectoderm, respectively (Kudoh et al., 2002). At the same time, gastrula cell movement occurs, changing the location of progenitors for the brain and spinal cord, and organizing the neural ectoderm to form the future neural plate arranged along the dorsal midline (Kudoh et al., 2004). The cell movements in gastrulation in zebrafish are categorized as convergence, extension, and epiboly movements (Figure 1B). Convergence is a cell movement by which laterally located cells move toward the dorsal axis. By extension, cells translocate the position along the animal pole to the vegetal pole (future anterior to posterior) axis, facilitating the elongation of the structures, including the neural plate and axial mesoderm. Besides these movements, there is an additional cell movement, epiboly. Epiboly is a critical cell movement during the gastrulation stage of fish embryos characterized by the formation and migration of multilayers of cells. The significant steps during epiboly include expanding enveloping and yolk syncytial layers, and cell re-arrangement between these two layers to thin the blastoderm and enveloping layer (e.g., Takesono et al., 2012). Epiboly cell movement occurs in all three germ layers (ectoderm, mesoderm, and endoderm), enveloping layer, and the yolk syncytial layer (Roszko et al., 2009). By combination of these 3 cell movements, cells at the gastrula are rearranged to locate to the final destination along the anterior brain to the posterior spinal cord.

When the gastrulation is complete, the newly formed neural plate is transformed to the neural tube by the process of neurulation, folding, and intercalation neural cell movement (Araya et al., 2016) (Figure 1C). At the same time, the eye field is formed at the anterior end of the neural plate, but it splits and migrates laterally to form two eyes (Figure 1C). This movement is induced and regulated by the morphogen, Sonic hedgehog (Shh), emanating from the underlying mesoderm (Ekker et al., 1995). Recent research works using zebrafish and other models revealed many of these processes are affected by ethanol, causing severe defects in the following development of the CNS. The details will be discussed in the following sections.

### Toxicity of Ethanol in Gastrulation

It has been shown in zebrafish that ethanol alters the gene expression and cell movement during the blastula stage and gastrula stage (Sarmah et al., 2020). Early ethanol exposure in zebrafish can severely affect gastrulation and epiboly cell movements (Blader and Strähle, 1998; Yelin et al., 2005; Zhang et al., 2010).

Sarmah et al., 2020 reported that the gene expression is already different before the gastrula stage in the ethanol-treated embryos they found that expression of sox2, which is a marker for stem cells and neural progenitor cells, is reduced in the ethanol-treated blastula stage embryos, leading to a significant delay in the epiboly at eight hpf. Injection of sox2 mRNA rescued the epiboly delay caused by the ethanol treatment, suggesting one of the key mechanisms of ethanol toxicity is suppression of stem cell factors such as sox2.

Blader and Strahle reported that ethanol exposure at the gastrula stage suppressed the migration of the chordal mesoderm, prechordal plate, causing failure of eye field separation and the cyclopia (Blader and Strähle, 1998). Zhang et al. (2010) showed that ethanol inhibited epiboly and convergence extension in the zebrafish embryos that are accompanied with gene expression changes: they reported that ethanol exposure altered gene expressions at the early gastrula stage that resulted in a scattered expression pattern of *chordin*, *wnt11*, and *eve1*, and delayed the migration of *gsc*-positive prechordal plate cells. The result indicates that the influence of ethanol on the gastrula cell movement is not restricted to one tissue but rather broadly affects the multiple cell fates and movement including the neural ectoderm and underlying mesoderm.

Sarmah et al. (2020) also found that the dorsal forerunner cells were closely linked to the germ ring in controls but significantly dissociated from the germ band after ethanol treatment of embryos, suggesting that there are differential sensitivities to ethanol in these tissues. It has been reported that ethanol also disrupts the microtubule cytoskeleton of the yolk syncytial layer, causing the suppressed microtubule filament formation, which is important for epiboly movement (Sarmah et al., 2020). Contractile actomyosin rings are involved in spreading of enveloping cell layer over the yolk cell during gastrulation. Ethanol-treated cells showed lamellipodia extension, which is a flattened extension of the actin filament in all directions, and cells in ethanol-treated embryos showed abnormal trajectories of animal and vegetal pole (Sarmah et al., 2013). The EVL cells in ethanol-treated embryos were round and not properly aligned, whereas in controls, these cells were elongated and aligned. High magnification images of stained control and ethanol-treated embryos revealed that only a few YSL nuclei in ethanol-treated embryos proceeded beyond the EVL, whereas all YSL nuclei in controls proceeded beyond EVL. Sarmah et al., 2013 evaluated cytoskeletal distribution and cell shapes by nuclear staining and actin. They found that actin-cytoskeleton associated with the enveloping layer was similar to control embryos at 90% epiboly. The data suggest that disruption of microtubule may alter cell polarity of the actin-mediated lamellipodia. If so, the key target of ethanol is possibly the microtubule rather than the actin cytoskeleton.

Ethanol also reduces the cell adhesion activity and cell directional movements during gastrulation. Cell movement at the gastrula stage is regulated by cell adhesion molecules such as Protocadherin 18a (pcdh18a) (Aamar and Dawid, 2008) and E-cadherin (Morita and Heisenberg, 2013). Sarmah et al., 2013 reported that ethanol exposure changed the distribution of E-cadherin, and it was redistributed into cytoplasmic aggregates in blastomeres and extraembryonic yolk cells. They further conducted the microarray analysis and showed that the pcdh18a expression was significantly reduced after the ethanol exposure. One possible epistatic mechanism might be that ethanol suppresses pcdh18a, causing the reduction of cell adhesion, altering protein localization of E-cadherin, and may cause depolymerization of the microtubule.

### Toxicity of Ethanol in CNS Morphogenesis

As discussed above, Blader and Strahle (1998) observed that the short exposure of 2.4% ethanol (dome to 30% epiboly) resulted in disrupted Wnt/PCP signaling resulting in the delay of anterior migration of the gsc-positive prechordal mesoderm. Prechordal mesoderm expresses Shh and split eye field to form two eyes. Therefore, the delay of the migration causes fused eyes and Cyclops phenotypes. The exposure of 3% ethanol for the same period resulted in a split body axis that is often linked with holoprosencephaly and cyclopia.

The ethanol exposure during zebrafish embryogenesis can also lead to morphological malformations. The reduction of eye diameter, body length, and pericardial edema has been reported in zebrafish embryos after the ethanol exposure (Joya et al., 2014), resulting in a significantly reduced eye size at 2.5% ethanol Zhang et al. (2013) have also presented that exposure to 0.5% ethanol can disrupt the mid-hindbrain boundary. Ethanol-induced defects in gastrulation can lead to the growth retardation of zebrafish. A disruption in the inner ear of embryos has been reported after treatment with 2% ethanol that may explain the human FASD symptoms related to the hearing loss (Zamora and Lu, 2013). Several other studies have also reported similar effects on zebrafish morphology after both binge and chronic ethanol treatments at 0.5–10% (Bilotta et al., 2004; Reimers et al., 2004; Ali et al., 2011; Zhang et al., 2013). Morphometric analyses have revealed that ethanol can change specific facial measurements, which might be due to higher cell death in the neural crest progenitors of the facial skeleton (Carvan et al., 2004; Flentke et al., 2014). All of these results indicate that ethanol can also affect cell movement and morphogenesis at the neurulation stage (Figure 1C), resulting in malformation of the eye formation, brain segmentation, and proper development of the neural tube.

Besides brain malformation, developmental abnormalities are also observed in the craniofacial structures in the neurula stage of the zebrafish embryos due to the abnormal development of the neural crest (Mccarthy et al., 2013). The neural crest from the mid-hindbrain area migrates anteriorly and forms the craniofacial structures. In the ethanol-treated zebrafish embryos, these cells fail to properly migrate to the final destination (Mccarthy et al., 2013). Besides migration and cell movement, it has also been reported that apoptotic cell death is induced in the neural tube and neural crest that further enhance the malformation of these structures (Mccarthy et al., 2013; Joya et al., 2014).

### Toxicity of Ethanol in Neurogenesis

Embryos of the transgenic zebrafish strain, Tg(HuC:KAEDE), express fluorescent protein in a range of neurons in the central and peripheral nervous systems. A decreased number of KAEDE-positive neurons in the spinal cord were found when embryos were exposed to 1% ethanol (Joya et al., 2014). Isl1 [Tg (Isl1:GFP) line] is a marker for developing motoneurons. Though the number of Isl1-positive motoneurons is not reduced, the study revealed that the motoneuron length in the embryos treated with ethanol was significantly decreased (47%) compared to the untreated controls (Joya et al., 2014). Several research groups have reported similar adverse effects of ethanol exposure on the motoneuron axonal branches and revealed a varied sensitivity of spinal motoneurons and cranial to ethanol (Sylvain et al., 2010, 2011; Coffey et al., 2013). Tg(Isl3:GFP) transgenic embryos express GFP in sensory neurons of the spinal cord and the sensory cranial ganglia, whereas Joya et al. depicted a significant decrease in the number of sensory neurons per somite (3.2 ± 1.1) as compared to the control treatment (5.8 ± 0.5). The TUNEL analysis of larval stages showed a higher apoptosis ratio in the embryo and the central nervous system (CNS). These results explain that ethanol exposure disturbs the balance between proliferation and apoptosis that reduces neuronal cell differentiation in a subset of ethanol-sensitive neurons (Joya et al., 2014). Zhang et al. (2013) reported high sensitivity of GABAergic and glutamatergic neuron development in the cerebellum or forebrain of zebrafish to ethanol. The phenotype of reduced neurogenesis was mimicked or enhanced by the suppression of gene expression of shh, fgfs, or agin, and the phenotype was also rescued by overexpression of the mRNAs encoding shh or a fgf. These results suggest that these signaling pathways are the targets of ethanol toxicity pathways, and modification of the pathway can reduce the ethanol toxicity.

### Link to Human FASD

Multiple factors contribute toward birth defects, but FASD caused by utero ethanol exposure has been thought to be the most common reason for abnormalities (Joya et al., 2014). A better understanding of the mechanisms involved in this syndrome would allow us to learn more subtle but still significant consequences of alcohol exposure and may also facilitate the development of clinical therapeutic interventions. In this regard, zebrafish has emerged as a model organism to explore environmental toxins related to congenital disability syndromes (Ali et al., 2011). As a vertebrate animal, zebrafish shares significant similarities with humans in the physiology, gene function, and organ development. FASD-related data retrieved from this zebrafish provide mechanical insights and a better understanding of the impacts of alcohol consumption by pregnant mothers. Zebrafish can mimic the ethanol-exposure–related developmental defects observed in humans, such as neural, craniofacial, and cardiac defects (Haycock, 2009; Kelly et al., 2009; Dlugos and Rabin, 2010; Muralidharan et al., 2013). During the early development, ethanol exposure could disrupt the developmental signaling mechanisms and affect the embryonic gene expression, cell movement, cell differentiation, and morphogenesis. The works from zebrafish have highlighted that exposure to alcohol at the early stage of embryo development has a crucial influence on later development of the brain. It has also been reported that even a moderate level of alcohol consumption can lead to hippocampal atrophy (Topiwala et al., 2017). This suggests that further research using model animals like zebrafish with a lower dose of exposure with long-term effects to the development and maintenance of brain would also become important.

### Advantages and Disadvantages of the Zebrafish as a Model for Human FASDs

The zebrafish provides an ideal model for studying the effects of alcohol as many phenotypes show similarities to human FASDs, including microcephaly, holoprosencephaly, spinal bifida, cyclopia, neural crest defects, craniofacial defects, and cardiac defects. Unlike human and mammalian models, it is possible to trace the development of living embryos being exposed to the controlled concentration of ethanol. It is also possible to screen drugs and mutations that can enhance/suppress the symptom of FASDs using high throughput large-scale screening. On the other hand, potential disadvantages may be that size and structure of the brain are much smaller and simpler than those of mammalian models; therefore, detailed symptom of FASDs may show some differences (e.g., gestation period–specific differences). As zebrafish develop externally without a gestation period, it is not possible to investigate the influence of the transfer of alcohol from the mother to fetus. It might also be conceivable that genetic mechanisms for metabolizing alcohol may have some variation between species, considering that even within the same mouse species, different strains show different sensitivity and symptoms to alcohol (Ogawa et al., 2005; Chen et al., 2011).

## Conclusion

FASD is a frequent birth defect syndrome that is related to alcohol consumption by pregnant mothers, affecting embryos and leading to birth defects. Research on zebrafish facilitates the investigation of ethanol-related defects during embryogenesis. It can be concluded that ethanol produces multifactorial defects during blastula, gastrula, and organogenesis stages, which are the key stages of embryogenesis. There are many target pathways of ethanol during the development including the stem cell gene expression, cytoskeletal regulation, and gastrulation cell movement, neurulation, eye morphogenesis, neural crest migration, and neurogenesis. With its sensitivity and simple morphology, it is possible to design high throughput testing and screening methods for assessing the dose-dependent effects of ethanol and other toxicants on brain development and associated human birth defects. It would also be important to investigate long-term effects of moderate dosage using zebrafish embryos to detect weak but significant effects to the development, growth, and behavior in the later life stages.
